# Variation in the susceptibility of *Anopheles gambiae* to botanicals across a metropolitan region of Nigeria

**DOI:** 10.1371/journal.pone.0210440

**Published:** 2019-01-09

**Authors:** Seun Olaitan Oladipupo, Amanda Callaghan, Graham J. Holloway, Olajire Ayodele Gbaye

**Affiliations:** 1 Department of Biology, Federal University of Technology, Akure, Nigeria; 2 Department of Entomology and Plant Pathology, Funchess Hall, Auburn University, Aurburn, Alabama, United States of America; 3 Centre for Wildlife Assessment and Conservation, School of Biological Sciences, University of Reading, Reading, United Kingdom; Universidade Federal do Rio de Janeiro, BRAZIL

## Abstract

Pesticide resistance is normally associated with genetic changes, resulting in varied responses to insecticides between different populations. There is little evidence of resistance to plant allelochemicals; it is likely that their efficacy varies between genetically diverse populations, which may lead to the development of resistance in the future. This study evaluated the response of *Anopheles gambiae* (larvae and adults) from spatially different populations to acetone extracts of two botanicals, *Piper guineense* and *Eugenia aromatica*. Mosquito samples from 10 locations within Akure metropolis in Southwest Nigeria were tested for variation in susceptibility to the toxic effect of botanical extracts. The spatial distribution of the tolerance magnitude (T.M.) of the mosquito populations to the botanicals was also mapped. The populations of *An*. *gambiae* manifested significant differences in their level of tolerance to the botanicals. The centre of the metropolis was the hot spot of tolerance to the botanicals. There was a significant positive correlation between the adulticidal activities of both botanicals and initial knockdown. Hence, knockdown by these botanicals could be a predictor of their subsequent mortality. In revealing variation in response to botanical pesticides, our work has demonstrated that any future use of botanicals as alternative environmentally friendly vector control chemicals needs to be closely monitored to ensure that resistance does not develop.

## Introduction

*Anopheles* mosquitoes are medically important because they are vectors of human diseases such as malaria, filariasis and arboviruses [1–2]. Due to a dearth of effective vaccines and drugs to control these diseases, attention has shifted towards the control of the mosquito disease vector [3], with a reliance on the use of synthetic insecticides [4]. However, concerns such as environmental toxicity, mammalian toxicity, effect on non-target species and insecticide resistance have long been associated with the use of synthetic insecticides [5–6].

An increase in the development of insecticide resistance in mosquitoes is of significant concern in all vector control programmes [7–10]. As alternatives, phytochemicals obtained from plants with proven insecticidal efficacy have been tested against mosquito vectors such as *Anopheles gambiae* [11–12]. Among the arrays of plant materials that have been reported to be effective in controlling this vector are extracts from the seeds of *Piper guineense* (Piperaceae) [12] and flower buds of clove *Eugenia aromatica* (Myrtaceae) [13]. *Piper guineense* is a West African spice plant, commonly called black pepper or Ashanti pepper that contains a cocktail of chemicals, including alkaloids and piperidene [14]. *Piper guineense* contains naturally-occurring piperine-type alkaloids [15] that have insecticidal properties against *Aedes aegypti*, *Culex quinquefasciatus* and *An*. *gambiae* [16–18]. Ethanolic extracts of *P*. *guineense* have also been used for mosquito control [13]. The active ingredient in clove is eugenol, a phenylpropene, which is known to exhibit biocidal properties toward *Sitophilus zeamais* [19], *Dinoderus bifloveatus* [20] and *Ixodes ricinus* [21]. In addition, the ethanolic extracts of *E*. *aromatica* can kill mosquito larvae within 24 h [22]. This suggests that applications of clove extracts could work rapidly during outbreaks and epidemics where immediate action is required.

Botanical pesticides have long been touted as attractive alternatives to synthetic chemical pesticides for mosquito control [23–25]. The possible reason for this is the development of resistance to virtually all classes of insecticides used for mosquito control [26–27]. However, it should not be assumed that naturally derived pesticides are less susceptible to resistance since they may well have similar modes of action. Thus far, history has shown that the overzealous use of synthetic pesticides has resulted in numerous problems that were unforeseen at the time of their introduction. Hence, tolerance surveys are useful to better understand insecticide tolerance patterns, explain control failures, and have a scientific basis for selection, rotation, and discontinuation of particular insecticides [28].

If we understand how the environment plays a role in the response of *Anopheles* mosquitoes to botanicals, we may be able to predict their tolerance to botanicals, and by extension, assist in the sustainable control of the vector. An alternative to synthetic insecticides is urgently required as current control of *Anopheles* mosquitoes in Southwest Nigeria using pyrethroid insecticides is being threatened by developing resistance [29–30]. Substantial evidence points to pyrethroid resistance [31–32] resulting from target-site mutations [33–34] and enhanced insecticide detoxification [35–36] with environmental factors influencing the mosquito responses to pyrethroids [33–38]. This study investigated the tolerance of *An*. *gambiae* within Akure metropolis in Southwest Nigeria to *P*. *guineense* and *E*. *aromatica* with a view to revealing probable locational differences and the likely hot spots of potential failure (if any) of botanical insecticide efficacy within the metropolis.

## Materials and methods

### Study area

**Fig 1:**
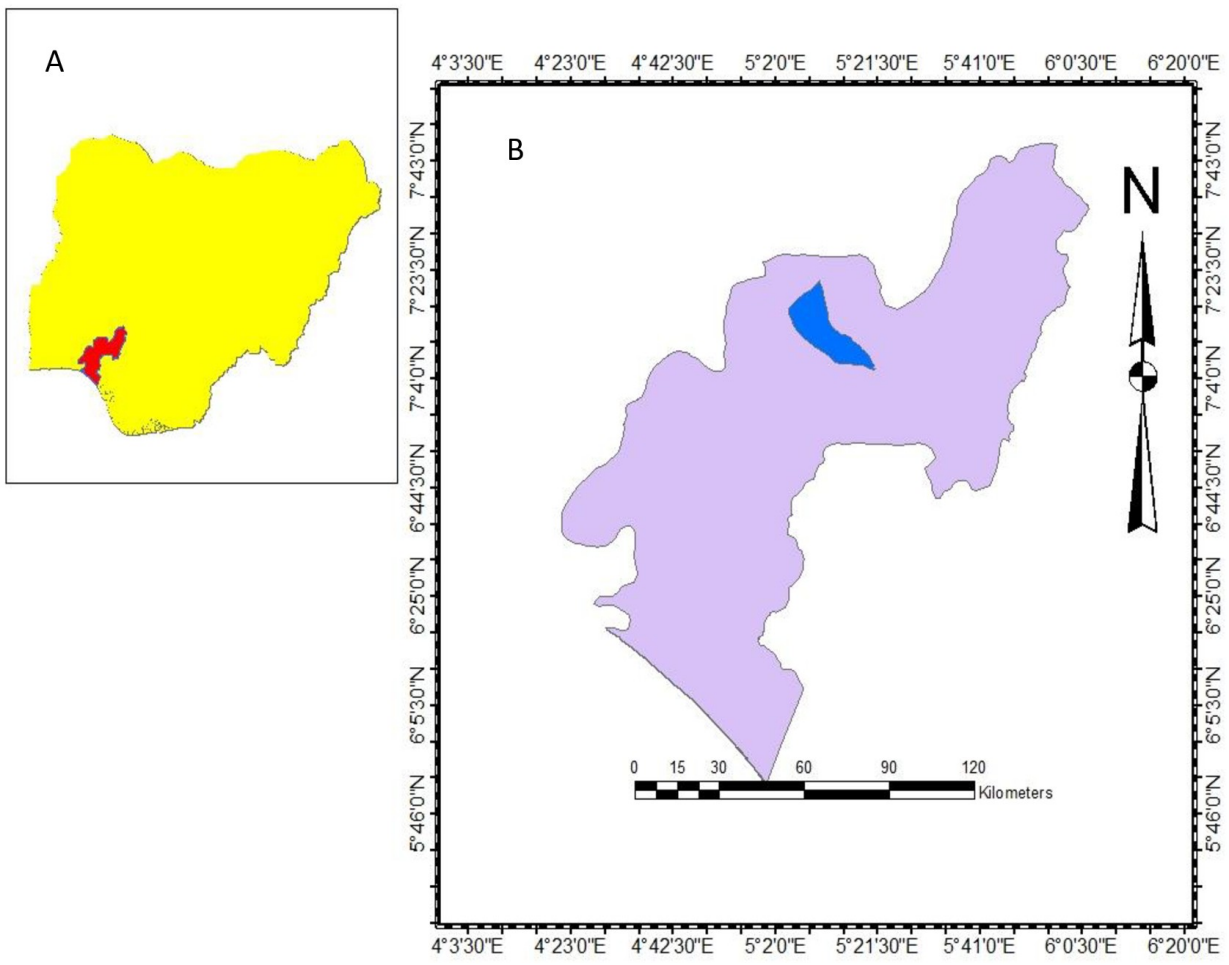
Map of (A) Nigeria showing Ondo State. (B) Ondo State showing Akure. The study area maps (Nigeria, Ondo State and Akure metropolis) were created by the authors using ArcGIS software (version 10.3). The shape files were obtained from the public online archive of maplibrary.org.

Akure (Fig 1) is the capital city of Ondo State in the South-Western region of Nigeria which is located at latitude 7.2571° N and longitude 5.2058° E of the equator, and situated at a mean elevation of 353 metres above sea level [39]. The climatic condition of Akure is influenced mainly by the rain-bearing southwest monsoon winds from the ocean and the dry northwest winds from the Sahara Desert. High temperatures and high humidity also characterize the climate [39].

### Botanicals

**Fig 2:**
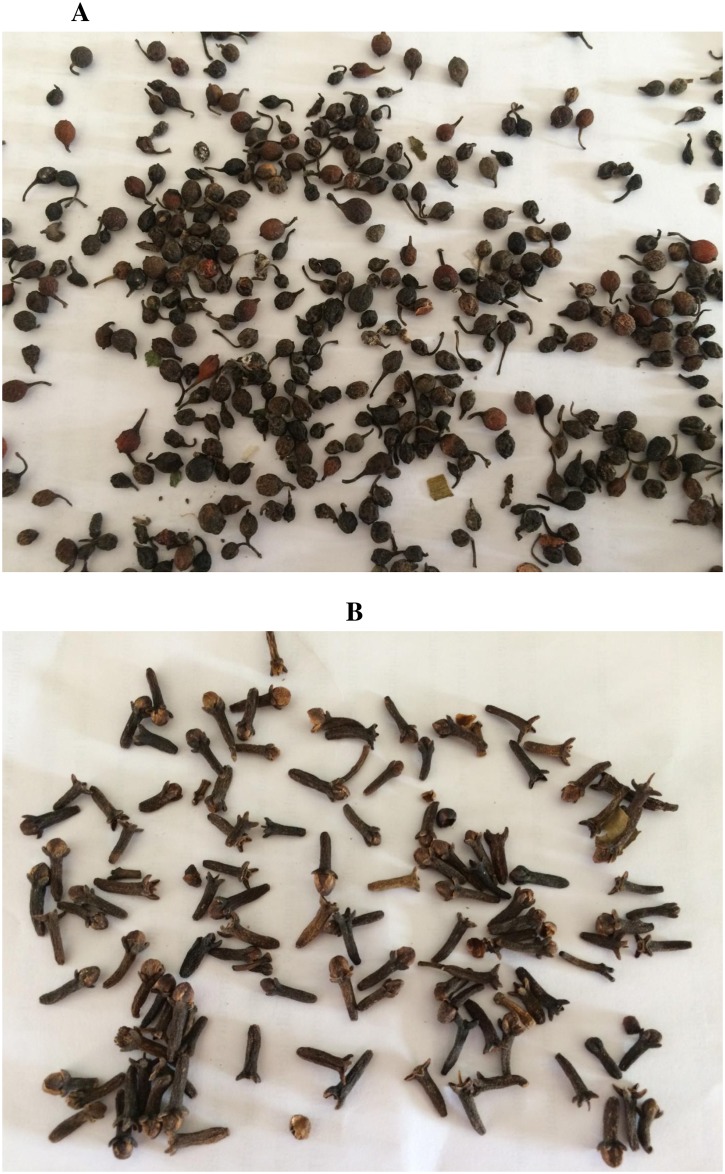
Seeds of *Piper guineense* (A) and dried flower buds of *Eugenia aromatica* (B).

The seeds of *P*. *guineense* (Fig 2A) and dried flower buds of *E*. *aromatica* (Fig 2B) were purchased from local herb sellers at Owena market (7.1965684N 5.0186405E) in Osun State, Nigeria. The plant materials were pulverized using a Marlex grinder (Model Excella 2431a, Marlex PVT LTD Mumbai, India). Their powders were sieved through a mesh size of 1mm^2^ and stored separately in tight lid containers at 28 ± 3°C and 75 ± 5% RH (relative humidity). Acetone extract of the botanicals was obtained separately using a cold extraction method [40]. This was done by soaking 300g of the powder in an extraction bottle containing 900ml of acetone for 72 hours. Filtration was then carried out using a double layer of Whatman No. 1 filter paper. The extraction solvent was evaporated using a rotary evaporator set at 35°C to 43°C with a rotary speed of 138 to 148 rpm for 3–4 hours. The resulting extracts were kept in bottles with tight lids and preserved in the refrigerator.

### Collection and rearing of mosquitoes

**Fig 3:**
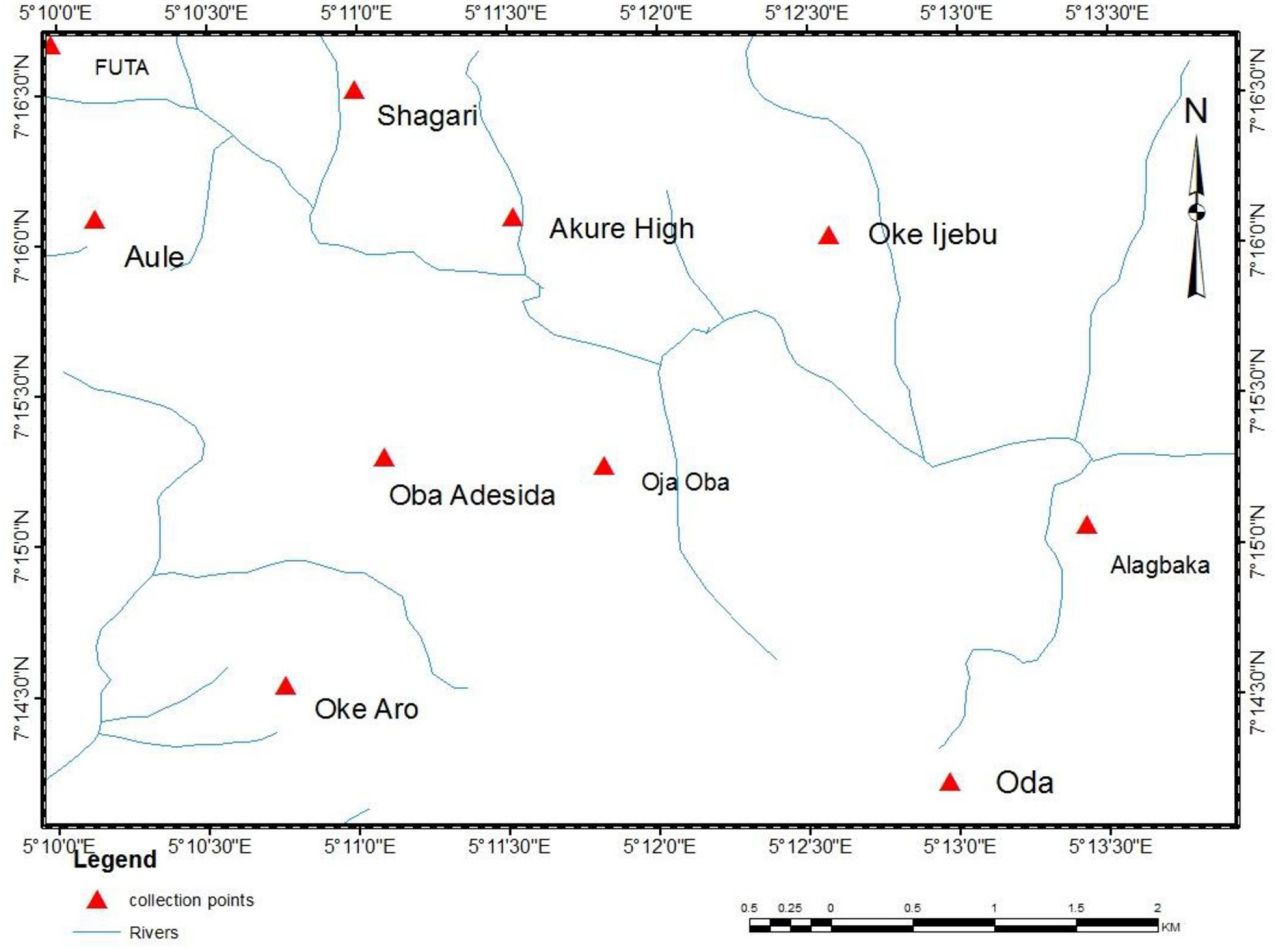
Sketch of Akure metropolis showing sample collection points of *Anopheles gambiae*.

*Anopheles gambiae* larvae were collected from 10 locations across Akure metropolis (Fig 3). The containers bearing mosquito larvae were transferred to the Entomology Laboratory of the Biology Department, Federal University of Technology, Akure, Nigeria. Larval identification was carried out under Olympus dissecting microscope (X20) using morphological keys [41, 42]. Once the larvae had pupated, they were transferred to a screened cage with dimension 20 x 20 x 20cm for adult emergence. The larvae were fed with yeast and reared at 28 ± 3°C and 75 ± 5% RH. The adult insects were used immediately after emergence.

#### Ethics statement

No specific permission was required for the collection activities. This is because the larvae collected, in each location, did not involve endangered or protected species. Also, mosquito larvae were collected from water bodies found within peri-domestic human surroundings and not from protected areas or private lands.

### Bioassay

#### Effect of plant extract on mosquito larvae

Larvicidal activities of the plant extracts were carried out according to a modified WHO standard procedure [43]. Fourth instar larvae of *An*. *gambiae* were used for this assay. After preliminary bioassays, 0.5ml of each concentration of *P*. *guineense* extract in acetone (0.1%, 0.3%, 0.4%, 0.5% and 0.7% delivering 0.0025μl/ml, 0.0075μl/ml, 0.0100μl/ml, 0.0125μl/ml and 0.0175μl/ml, respectively) was added to 200ml water in 250ml glass beaker. Also, 0.5ml of each concentration of *E*. *aromatica* extract in the solvent (2%, 3%, 4%, 5% and 6% delivering 0.050μl/ml, 0.075μl/ml, 0.100μl/ml, 0.125μl/ml and 0.150μl/ml, respectively) was used for the bioassay. Twenty mosquito larvae were used for each assay and four replicates were set up for each concentration and the control (0%). The control beaker contained only 0.5ml of acetone in water. Larval mortality was observed after 24 hours [44]. Larvae that failed to respond to prodding or make it to the surface for respiration were judged to be dead. The assay described above was performed separately using extracts from both botanicals on all the sampled populations.

#### Fumigant effect of plant extracts on adult mosquito

The fumigant effect of the plant extracts against adult mosquitoes was assessed by using a modified WHO protocol [45] impregnated filter paper technique [46]. The base of a 250ml plastic container (diameter—50mm (upper) and 35mm (lower); height—90mm) was cut open and clogged with cotton wool. The top of the container was covered with muslin cloth fastened with a rubber band. Strips of Whatman’s No.1 filter papers (3cm x 3cm) were doused with 0.3 ml of varying concentrations of the plant extracts in acetone (*P*. *guineense* [0.7%, 0.9%, 1.2%, 1.4% or 1.6% delivering 0.0084μl/cm^3^, 0.0108μl/cm^3^, 0.0144μl/cm^3^, 0.0168μl/cm^3^ or 0.0192μl/cm^3^, respectively] and *E*. *aromatica* [6%, 7%, 8%, 9% or 11% delivering 0.072μl/cm^3^, 0.084μl/cm^3^, 0.096μl/cm^3^, 0.108μl/cm^3^ or 0.132μl/cm^3^, respectively]. The solvent was allowed to evaporate from the strip before it was placed below the cotton wool in cut part of the container to avoid contact with the mosquitoes. Ten adult *An*. *gambiae* were introduced into each plastic container using a pooter. Each treatment and the control (filter paper treated with 0.3 ml of acetone only) were replicated three times. The assays for the two botanicals were done separately to avoid synergistic effects. Adults were exposed to treatments for 60 minutes before the removal of the treated filter paper and assessment of knockdown. A mosquito was considered knocked down if it lay on its side on the floor of the container and was unable to fly. Mortality was recorded 24 hours post exposure period.

### Data analysis

The data were arcsine transformed and subjected to probit analysis [47] to determine the median lethal dose (LD_50_) and the median knockdown dose (KD_50_) of both botanical insecticides for each mosquito population. General Linear Modelling (GLM) was used to examine global differences among mosquito populations and treatment means at P < 0.05. Where significant differences occurred, the means were separated using Tukey’s post-hoc test. The main effects of location (L) and concentration (C) and their interaction (LxC) on the tolerance of *An*. *gambiae* to both mortality and knockdown effect of the botanicals were analyzed.

The relationship between knockdown (KD_50_) and mortality (LD_50_), for each population and botanical was investigated using Pearson correlation matrix. Given that different chemical compounds are present in the botanicals, correlation of *An*. *gambiae* tolerance between the botanicals for each population was also investigated. All analyses were carried out using Statistical Package for Social Sciences (SPSS) version 20. The tolerance magnitude (T.M.) of the mosquito populations (from each location for each plant extract) was calculated using the following expression:

$$T.M.=\frac{{LD}_{50}\;of\;each\;location}{{Highest\;LD}_{50}}\times \frac{360}{1}$$

[48]

The spatial distribution of T.M. of the mosquito populations was mapped and plotted using ArcMap10.3 software.

## Results

### Larval LD_50_

Irrespective of the plant extract used, generally Alagbaka mosquito populations were the most susceptible, with the lowest LD_50_. The Oke-Aro larvae population had the highest LD_50_ when exposed to the *P*. *guineense* extract (0.56%) while the Oja-Oba population had the highest LD_50_ when exposed to *E*. *aromatica* extract (6.09%) (Table 1). For the homogeneity of response (slope of the log-dose probit relationship), the Oba-Adesida population had the steepest slope for *P*. *guineense* (4.12) and *E*. *aromatica* (4.27). Shagari population had the shallowest slope for *P*. *guineense* (1.50) and FUTA had the shallowest slope for *E*. *aromatica* (1.93). For other populations, the slope values ranged from 2.39 to 3.06 for *P*. *guineense* and 2.47 to 3.59 for *E*. *aromatica*.

**Table 1:** Lethal dose (%) of botanical insecticides required for the mortality of *Anopheles gambiae* larvae in Akure metropolis.

LOCATION	*P*.*guineense*		LD_50_ (95% FL)	*E*. *aromatica*		LD_50_ (95% FL)
	Slope (±S.E)	Intercept (±S.E)		Slope (±S.E)	Intercept (±S.E)	
**Oba-Adesida**	4.12 (±1.57)	5.63 (±1.56)	0.04 (0.03–0.06)	4.27 (±0.21)	1.56 (±0.11)	2.32 (1.79–2.70)
**Akure High**	2.39 (±0.74)	-0.42 (±0.79)	0.04 (0.01–0.04)	3.08 (±0.23)	1.39 (±0.12)	2.83 (2.31–3.27)
**Alagbaka**	**	**	**	3.59 (±0.48)	0.16 (±0.20)	1.11 (0.12–1.61)
**Oke-Aro**	2.56 (±0.18)	-1.92 (±0.23)	0.56 (0.21–0.86)	3.11 (±0.19)	1.11 (±0.11)	2.27 (1.94–2.54)
**Aule**	**	**	**	2.47 (±0.33)	0.35 (±0.15)	1.38 (0.34–1.89)
**Oja-Oba**	2.98 (±0.16)	4.97 (±0.25)	0.47 (0.42–0.53)	2.62 (±0.18)	0.91 (±0.10)	6.09(5.37–7.41)
**Oda**	2.49 (±0.14)	-2.53 (±0.19)	0.10 (0.06–0.14)	2.84 (±0.20)	0.77 (±0.11)	2.20 (1.77–2.53)
**Oke-Ijebu**	3.37 (±0.92)	-1.85 (±0.94)	0.05 (0.01–0.05)	3.36 (±0.20)	1.15 (±0.11)	2.40 (2.09–2.65)
**Shagari**	1.50 (±0.24)	-0.10 (±0.30)	0.01 (0.01–0.03)	3.07 (±0.19)	1.17 (±0.11)	1.87 (1.93–2.47)
**FUTA**	3.06 (±1.90)	5.24 (±1.88)	0.02 (0.02–0.03)	1.93 (±0.33)	0.52 (±0.16)	0.54 (0.01–1.26)

S.E: Standard error; FL: Fiducial limits; LD: Lethal dose;

** = Figures could not be computed due to total larvae mortality caused by some of the experimental concentrations.

### Adult KD_50_ and LD_50_

For the adult knockdown assay, Oja-Oba population had the highest KD_50_ when exposed to *P*. *guineense* extract (1.93%) (Table 2) and *E*. *aromatica* extract (KD_50_ = 8.40%) (Table 3). However, Aule population had the steepest slope (5.60) (Table 2) for *P*. *guineense*, indicating high homogeneity of the population while Oda population had the shallowest slope (2.03). For *E*. *aromatica*, FUTA had the steepest slope (19.77) and Shagari had the lowest slope (5.86).

**Table 2:** KD_50_ (%) and LD_50_ (%) of *Piper guineense* required for *Anopheles gambiae* adult populations in Akure metropolis.

LOCATION	Slope (±S.E)	Intercept (±S.E)	KD_50_ (95% FL)	Slope (±S.E)	Intercept (±S.E)	LD_50_ (95% FL)
**Oba-Adesida**	5.28 (±0.29)	-0.30 (±0.04)	1.14 (1.07–1.22)	3.84 (±0.34)	-1.15 (±0.05)	1.99 (1.69–2.78)
**Akure High**	5.51 (±0.29)	-0.42 (0.04)	1.19 (1.11–1.28)	6.71 (±0.55)	-1.19 (±0.08)	1.51 (1.39–1.74)
**Alagbaka**	4.04 (0.37)	0.72 (±0.04)	0.66 (0.42–0.79)	2.83 (±0.26)	0.20 (±0.04)	0.85 (0.63–0.99)
**Oke-Aro**	4.02 (0.27)	0.09 (0.04)	0.95 (0.85–1.04)	3.21 (0.28)	-0.54 (±0.04)	1.47 (1.33–1.74)
**Aule**	5.60 (±0.29)	-0.06 (±0.04)	1.02 (0.89–1.15)	4.68 (±0.33)	-0.96 (±0.05)	1.60 (1.40–2.11)
**Oja-Oba**	4.01 (±0.41)	-1.15 (0.06)	1.93 (1.65–2.81)	3.07 (±0.48)	-1.17 (±0.07)	2.41 (2.06–3.20)
**Oda**	2.03 (±0.26)	0.33 (±0.04)	0.69 (0.53–0.79)	2.86 (±0.28)	-0.75 (±0.04)	1.83 (1.52–2.88)
**Oke-Ijebu**	5.01 (±0.28)	-0.15 (±0.04)	1.07 (0.92–1.23)	4.89 (±0.31)	-0.83 (±0.04)	1.47 (1.29–1.91)
**Shagari**	3.88 (±0.27)	-0.08 (±0.04)	1.05 (0.96–1.13)	4.49 (±0.32)	-0.95 (±0.05)	1.63 (1.44–2.07)
**FUTA**	2.43 (±0.29)	0.71 (±0.04)	0.51 (0.16–0.65)	2.23 (±0.26)	0.12 (±0.04)	0.89 (0.40–1.11)

S.E: Standard error; FL: Fiducial limits; KD: Knockdown dose; LD: Lethal dose

As observed with the KD_50_, Oja-Oba population had the highest LD_50_ values for both botanicals, 2.41% and 11.50% for *P*. *guineense* and *E*. *aromatica*, respectively (Tables 3 and 4). For *P*. *guineense*, Akure High population had the steepest slope (6.71) and FUTA population recorded the shallowest slope (2.23). For *E*. *aromatica*, FUTA had the steepest slope (16.75) while the Oba-Adesida population had the shallowest slope (2.82).

**Table 3:** KD_50_ (%) and LD_50_ (%) of *Eugenia aromatica* required for *Anopheles gambiae* adult populations in Akure metropolis.

LOCATION	Slope (±S.E)	Intercept (±S.E)	KD_50_ (95% FL)	Slope (±S.E)	Intercept (±S.E)	LD_50_ (95% FL)
**Oba- Adesida**	13.01(±0.81)	-10.40 (±0.67)	6.30 (5.95–6.56)	2.82 (±0.37)	-2.56 (±0.33)	8.26 (7.02–10.12)
**Akure High**	6.69 (±0.67)	-4.95 (±0.57)	5.48 (4.29–6.07)	5.87 (±0.42)	-5.00 (±0.37)	7.13 (5.76–8.00)
**Alagbaka**	**	**	**	9.86 (±0.96)	-7.11 (±0.80)	5.26 (3.96–5.82)
**Oke-Aro**	4.49 (±0.42)	-3.35 (±0.37)	5.58 (4.42–6.21)	4.71 (±0.38)	-4.60 (±0.36)	9.46 (8.58–11.28)
**Aule**	5.99 (±0.70)	-4.22 (±0.60)	5.07 (0.15–6.12)	3.80 (±0.37)	-3.40 (±0.34)	7.85 (6.86–8.86)
**Oja-Oba**	8.09 (±0.43)	-7.48 (±0.40)	8.40 (7.97–8.84)	3.84 (±0.41)	-4.07 (±0.37)	11.50 (9.82–19.45)
**Oda**	12.39(±0.76)	-9.99 (±0.64)	6.41 (6.10–6.64)	11.62 (±0.54)	-10.25 (±0.48)	7.63 (7.43–7.82)
**Oke-Ijebu**	8.68 (±0.86)	-6.22 (±0.72)	5.21 (4.01–5.78)	10.23 (±0.70)	-8.18 (±0.60)	6.31 (5.39–6.79)
**Shagari**	5.86 (±0.80)	-3.82 (±0.68)	4.49 (0.25–5.63)	5.37 (±0.63)	-3.96 (±0.54)	5.46 (2.85–6.28)
**FUTA**	19.77(±2.01)	-14.92 (±1.61)	5.67 (5.04–5.96)	16.75 (±1.20)	-13.23 (±0.98)	6.16 (5.78–6.42)

S.E: Standard error; FL: Fiducial limits; KD: Knockdown dose; LD: Lethal dose

** = Figures could not be computed due to total knockdown by some of the experimental concentrations.

**Table 4:** Relationship between the activities of *Piper guineense* and *Eugenia aromatica* on *Anopheles gambiae*.

Botanicals	Investigated index	r*	P-value**
*P*. *guineense*	Larvae mortality vs Adult mortality	0.14	0.06
	Adult knockdown vs Adult mortality	0.81	0.01
*E*. *aromatica*	Larvae mortality vs Adult mortality	0.61	0.30
	Adult knockdown vs Adult mortality	0.82	0.01
*P*. *guineense* vs *E*. *aromatica*	*P*. *guineense* L.LD vs *E*. *aromatica* L.LD	0.57	0.14
	*P*. *guineense* KD vs *E*. *aromatica* KD	0.61	0.08
	*P*. *guineense* A.LD vs *E*. *aromatica* A.LD	0.75	0.01

L.LD = Larvae LD_50_; KD = Adult KD_50_; A.LD = Adult LD_50_

*r = Correlation coefficient;

** Significance level is at P < 0.05

### Effect of location and concentration on susceptibility

#### Larvae

GLM revealed a highly significant effect of location (L) on the susceptibility of *An*. *gambiae* larvae to *P*. *guineense* (F_9, 177_ = 2441.18 p < 0.0001) and *E*. *aromatica* (F_9, 177_ = 614.88, p < 0.0001). It also revealed a highly significant effect of concentration (C) on the susceptibility of *An*. *gambiae* larvae to *P*. *guineense* (F_5, 177_ = 182.85, p < 0.0001) and *E*. *aromatica* (F_5, 177_ = 96.94, p<0.0001). There were significant interactions between location and concentration on the susceptibility of the larvae to *P*. *guineense* (F_45, 177_ = 187.68 p< 0.0001) and *E*. *aromatica* (F_45, 177_ = 93.89, p< 0.0001).

#### Adults

In contrast to the larval results, GLM revealed no significant effect of location (L) on the susceptibility of *An*. *gambiae* adults to knockdown and mortality effects of the botanicals. It however showed a significant effect of concentration (C) of the botanicals on the vectors susceptibility to both knockdown (*P*. *guineense*: F_5, 118_ = 3.19, p = 0.01; *E*. *aromatica*: F_5, 118_ = 2.39, p = 0.04) and mortality (*P*. *guineense*: F_5, 118_ = 2.80, p = 0.02; *E*. *aromatica*: F_5, 118_ = 2.88, p = 0.02). There was no significant interaction between location and concentration on the susceptibility of *An*. *gambiae* adults to knockdown and mortality to either botanical.

### Relationship between the activities of *P*. *guineense* and *E*. *aromatica*

The relationships between the activities of the tested botanicals on *An*. *gambiae* are shown in Table 4. There were positive correlations between the larvicidal and adulticidal activities of *P*. *guineense* (r = 0.14; p = 0.06) and *E*. *aromatica* (r = 0.61; p = 0.30) on *An*. *gambiae*. However, the correlations were not significant. There were significant positive correlations between adult knockdown and mortality effected by both botanicals (*P*. *guineense*: r = 0.81, p = 0.01; *E*. *aromatica*: r = 0.82, p = 0.01) (Table 4).

There was a positive but non-significant correlation between the larvicidal activities of the two botanicals (Table 4). The adult knock-down activities of the botanicals was also positive but not significant. However, there was a significant positive correlation (r = 0.75; p = 0.01) between the adulticidal activities of the botanicals.

### Spatial variation in tolerance magnitude (T.M.)

**Fig 4:**
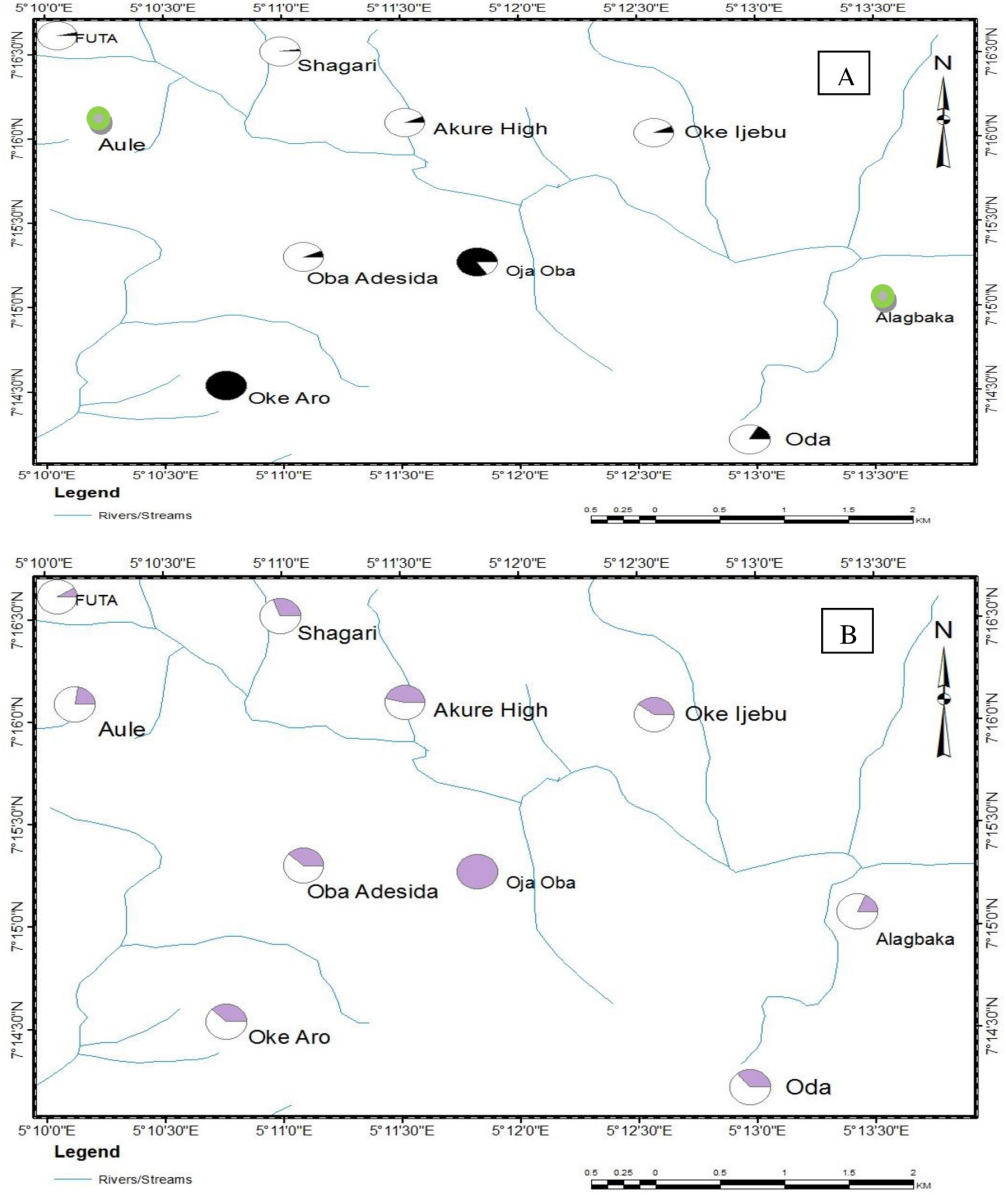
Map of Akure metropolis showing the spatial variation in tolerance magnitude of *Anopheles gambiae* larvae to (A) *Piper guineense* (B) *Eugenia aromatica*. *The larger the shaded area on a pie, the greater the tolerance. **Green circles indicate TM could not be calculated due to the inability to compute the LD_50_ value.

**Fig 5:**
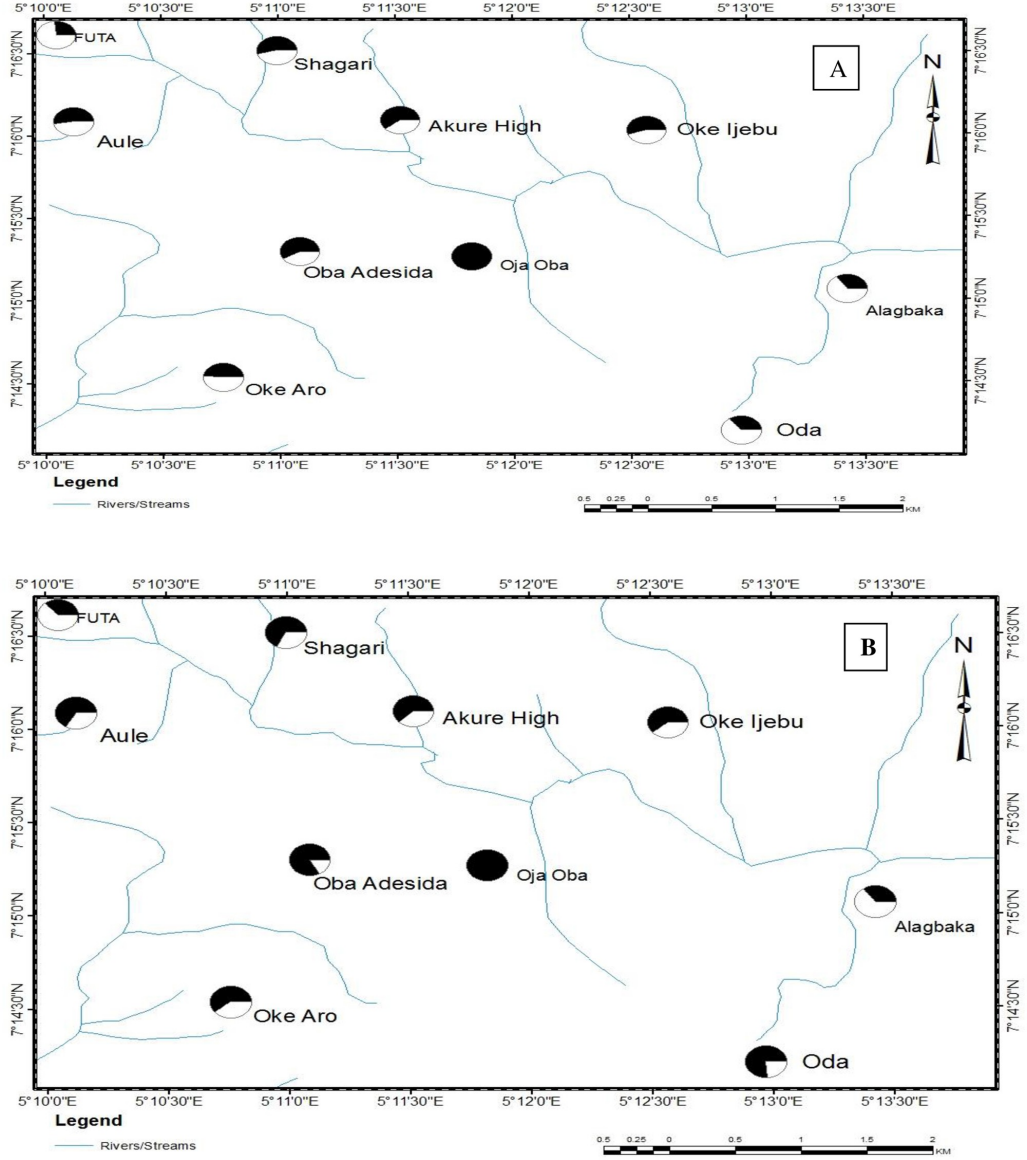
Map of Akure metropolis showing the spatial variation in tolerance magnitude of adult *Anopheles gambiae* to (A) knockdown effect and (B) mortality effect of *Piper guineense*. *The larger the shaded area on a pie, the greater the tolerance.

**Fig 6:**
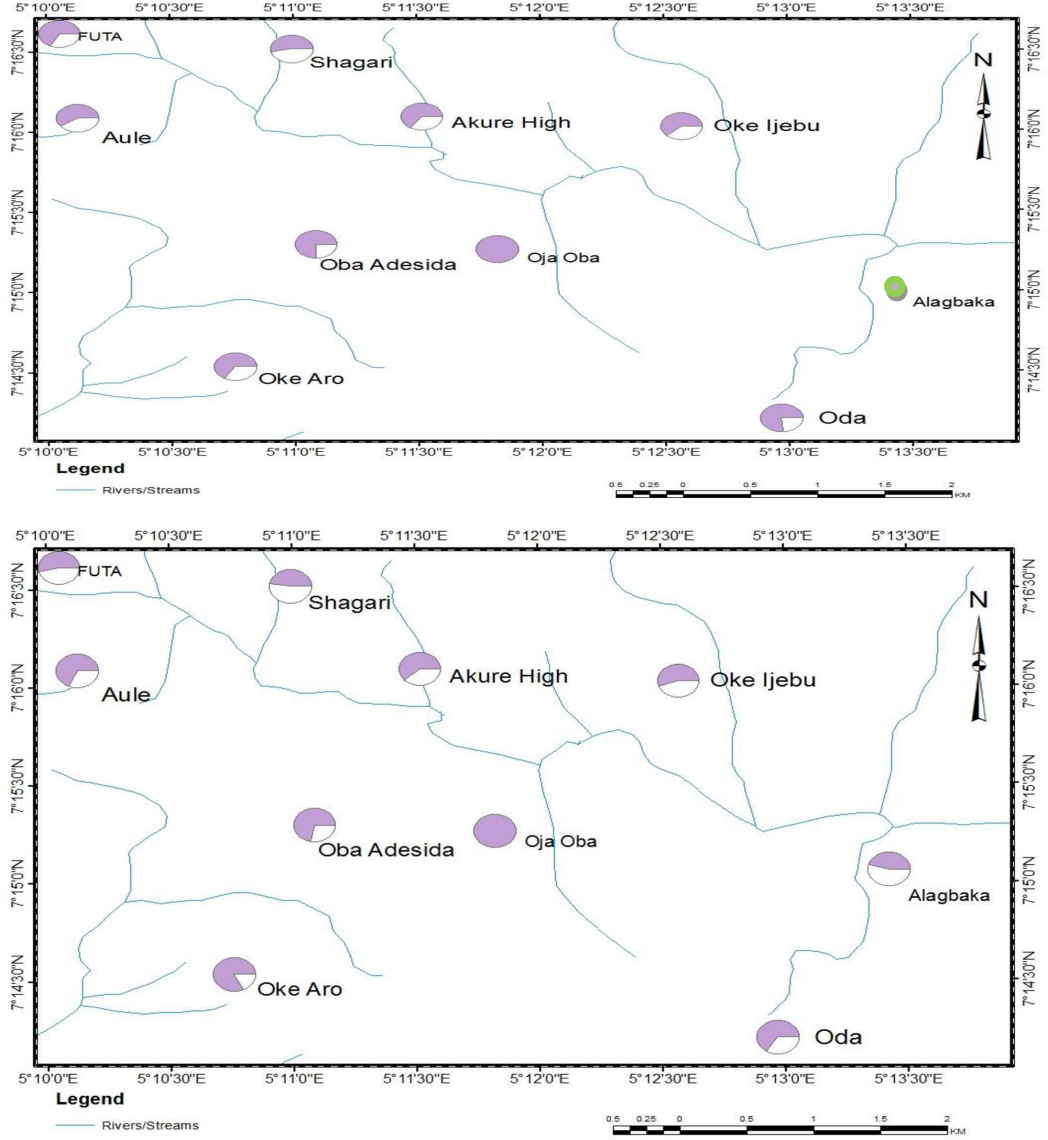
Map of Akure metropolis showing the spatial variation in tolerance magnitude of adult *Anopheles gambiae* to (A) knockdown effect and (B) mortality effect of *Eugenia aromatica*. *The larger the shaded area on a pie, the greater the tolerance. **Green circles indicate TM could not be calculated due to the inability to compute the KD_50_ value.

The spatial distribution of T.M. of *An*. *gambiae* larval populations in Akure metropolis to botanical insecticides is shown in Fig 4. In relation to Oke-Aro (the location with the highest T.M.) in the south west of the region, generally low larval susceptibility to *P*. *guineense* was observed across the metropolis (Fig 4A). The only exception was seen in the centre of the metropolis where the Oja-Oba population had a T.M. about three quarters that of Oke-Aro (Fig 4A). Similarly, for *E*. *aromatica*, a low tolerance magnitude was seen across the metropolis. Mosquito larvae from the centre of the town (Oja-Oba) exhibited the highest tolerance in comparison to the other populations (Fig 4B). The Oja-Oba *An*. *gambiae* adult population had the highest level of tolerance to both the knockdown and mortality effect of the botanicals. This identified Oja-Oba as the tolerance hotspot region (Figs 5 and 6). The FUTA population in the Northwestern part of the metropolis had a KD_50_ only one quarter that of the highest KD_50_ (Oja-Oba) (Fig 5A) while the Alagbaka and Oda populations in the East and South East, respectively, had KD_50_ values only one third that of the Oja-Oba population. Moderate levels of more than half of Oja-Oba tolerance were observed across the metropolis.

## Discussion

This study found significant differences in the tolerance of larvae and adult *An*. *gambiae* to two botanical preparations with putative insecticidal properties. There are at least two reasons why the susceptibility of larvae and adults is different. Larvae are filter feeders and therefore ingesting the compound, whilst in adults the compounds have to penetrate the insect through the cuticle. Once in the gut the compounds could be actively taken into the body along with nutrients; the compounds are most likely passing passively through adult cuticle. We have no idea how or whether the active ingredients are modified or de-activated differentially between larvae and adults. The second issue is that the compounds are delivered in different ways for larvae and adults. Again, we do not know whether mode of delivery affects the active compounds in any way.

Alagbaka populations of *An*. *gambiae* were the most susceptible whereas the Oja-Oba populations of *An*. *gambiae* adults were the most tolerant to the botanicals. These differences could be due to local environmental conditions. The abundance of susceptible individuals of *An*. *gambiae* in an area is sometimes due to the availability of unpolluted breeding sites [49]. It is possible that the presence of susceptible individuals in this area (Alagbaka) is influenced by the availability of unpolluted ground water pools and environmental conditions that encourage rapid development of the mosquitoes. Alagbaka is a Government Reserved Area (G.R.A) in the Akure metropolis. It is a residential area that is characterized by a proper drainage system and waste management. In contrast, Oja-Oba is located centrally in Akure and boasts the biggest market in the metropolis. The wastes or by-product of plant materials are washed into the gutters, some of which are stagnant. Therefore, these water bodies might have been polluted with defensive chemical compounds from these plant sources, thus enhancing the tolerance of *An*. *gambiae* to the botanicals investigated. Kim and Muturi [50] examined the relationship between mosquito species reared on leaf litters and induction of cytochrome genes (CYP450). Their result demonstrated that the genes were induced enhancing the metabolism of toxic products that the mosquitoes were exposed to. Other studies have also reported the role of natural xenobiotics in boosting mosquito larvae response to insecticides [51– 52]. Considering that the mosquito breeding sites in Oja-Oba contain dissolved plant chemicals or plant particles, the question of the relative impact of these natural xenobiotics on the response of mosquitoes is pertinent [50]. Their presence might affect mosquito metabolism, modifying their tolerance to insecticides. This might increase selection pressure and lead to the development of biocidal resistance. It is commonly found that resistance occurs through the production of detoxifying enzymes that degrade insecticides before they are able to exert their effect [27, 53–55]. This phenomenon has been identified in mosquito populations for all major classes of insecticides such as organophosphates and pyrethroids [53–55]. Potent botanicals present in an environment due to indiscriminate discarding (as in Oja-Oba) or use for agricultural, economic and other associated purposes, could stimulate the development of resistance. Differences between populations could reflect the local breeding sites, so that mosquitoes that bred in water into which plant material fell (allowing their chemicals to leach into the water) could be better adapted to cope with botanicals [56]. In Southwest Nigeria, as elsewhere, vector control programmes rely exclusively on the application of chemical insecticides, especially pyrethroids, either through the use of spraying or in insecticide treated nets. The control of agricultural and urban insect pests in this region also relies heavily on both synthetic pyrethroids and organophosphates. Nkya *et al* [33] argued that the presence of natural xenobiotics in mosquito breeding sites and pesticide usage in agriculture influences mosquito response to pyrethroids.

GLM revealed a highly significant effect of location on the susceptibility of *An*. *gambiae* larvae to the two botanicals used. This is in contrast to the adult stage where GLM revealed no locational effect on both knockdown and mortality. However, a closer look at the spatial variation of T.M. shows locational differences in the response of the adults of the various sampled populations of *An*. *gambiae* to the botanicals used. This study revealed that areas with similar habitat characteristics had similar tolerance level, thus suggesting that environmental factors might influence the among population tolerance differences noted here. For instance, the Oba-Adesida and Oja-Oba, areas are characterized by dirtier and more polluted breeding sites. Mosquito populations from these sites exhibited higher levels of tolerance to the botanicals investigated. In contrast, the Alagbaka and FUTA populations of *An*. *gambiae* were collected from cleaner water and had higher susceptibility levels. Assaying synthetic pyrethroid and organophosphate on the same *An*. *gambiae* populations from the studied areas; it was also observed that some of the implicated areas showed some tolerance similarities to what was obtained with botanicals (Gbaye and Oladipupo, pers comm). The differences in the tolerance magnitude of *An*. *gambiae* populations to the botanical insecticides could be linked to the condition of their breeding sites. Brittany *et al*. [57] reported that the conditions experienced by the larval population of *An*. *gambiae* play a key role in adult susceptibility to insecticides. According to Aguirre-Obando *et al*. [58], *Aedes aegypti* populations sourced from diverse locations in Brazil showed variations in their level of susceptibility to insecticides used. Similarly, Polson *et al*. [59], reported that Cambodian populations of *A*. *aegypti* larvae manifested variable differences in their level of susceptibility to a synthetic insecticide depending on whether or not they had prior exposure to similar chemicals in their environment. Thus, the knowledge of the effects of environmental conditions on immature mosquito development is important for the inference of results of laboratory experiments on mosquito tolerance.

A significant positive correlation between the adulticidal activities of *P*. *guineense* and *E*. *aromatica* on *An*. *gambiae* populations was observed in this study. This implies that an adult population of *An*. *gambiae* susceptible to *P*. *guineense* might also be susceptible to *E*. *aromatica*. The components of these two botanicals are different and there is not a priori reason why we would expect the insects to respond to them in exactly the same way. Variation in the larval breeding site and also in the genetic background of the population could affect the mosquitoes’ response. There exists potential variation in the activities of the used botanicals and/or titres of detoxification enzymes in different mosquito populations which have most likely evolved to deal with plant chemicals [60]. There is the need to increase the number of botanical types to ascertain the universality of this observation. There were also positive correlations between adult knockdown and mortality caused by each botanical. This is in line with the study of Norris *et al* [61], they also observed correlation between *An*. *gambiae* knockdown and mortality caused by several botanical oils {which also include clove bud}. Same cannot be said for *Aedes aegypti* in their study, hence this observation might be species or genus dependent. It implies that the susceptibility of an *An*. *gambiae* population to knockdown by the plant extracts could be a predictor of the mortality caused by the same extract. This contradicts the findings of Owusu *et al* [62] who argued that knockdown by synthetic insecticide is a poor predictor of 24 h mortality. Although our study is on botanicals with a cocktail of (probable) synergistic chemicals, Owusu *et al* [62] were actually comparing the 24h mortality in WHO assay with knockdown in the CDC (Center for Disease Control and Prevention) bottle assay. The mix of several active components in botanicals, such as *P*. *guineense* and *E*. *aromatica*, might have prevented the recovery of several of the *An*. *gambiae* populations after knockdown in our study. Feng and Isman [63] argued that a cocktail of active components from the whole of a botanical insecticide is able to deter resistance development better than a single isolated plant compound.

Larvicidal activity of *E*. *aromatica* against *An*. *gambiae* was observed to have a significant positive correlation with its adulticidal activity unlike *P*. *guineense* (positive but relationship not significant). This indicates that any population susceptible to *E*. *aromatica* at the larval stage might also be susceptible at the adult stage. Such a relationship is not found with all synthetic insecticides, for example bendicarb (a carbamate) where a negative relationship between *An*. *gambiae* larval and adult mortality was found [64].

The centre of the metropolis studied seems to be the hotspot of tolerance to the botanicals investigated. This might be an indicator of probable sites of control failure with any future use of botanicals. There is a need for further work on populations from these locations with regard to enzymatic activity to further corroborate the findings in this study. Although a standardized method of testing botanical efficacy remains to be settled on [65], there is a clear need for wider research to establish the level of tolerance to known botanicals. Likewise, further investigation on the influence of environmental factors on the response of insects to botanicals is paramount to enhance and sustain the development and application of botanical insecticides.

## Conclusion

This study revealed that (1) in areas within the metropolis with similar habitat characteristics, *An*. *gambiae* had similar tolerance level to botanicals; (2) the susceptibility of an *An*. *gambiae* adult population to knockdown by a botanical could be a predictor of the mortality caused by the same plant; (3) a population of *An*. *gambiae* susceptible to *E*. *aromatica* at the larval stage might also be susceptible at the adult stage; (4) spatial analysis implicated the centre of the metropolis studied to be the hotspot of tolerance to the botanicals investigated, hence, an indicator of probable site of control failure with the future use of the botanicals.

## Supporting information

S1 File Data points for An gambiae botanicals. (XLSX)
